# Heterogeneity of Urban and Rural Areas in Association of Fringe Benefits and Depression: A Cross-Sectional Study

**DOI:** 10.3389/fpubh.2022.811165

**Published:** 2022-01-31

**Authors:** Tianwen Luo, Chaoyang Yan, Xiang Zhang

**Affiliations:** ^1^Department of Health Management, School of Medicine and Health Management, Tongji Medical College, Huazhong University of Science and Technology, Wuhan, China; ^2^The Key Research Institute of Humanities and Social Science of Hubei Province, Huazhong University of Science and Technology, Wuhan, China

**Keywords:** depression, fringe benefits, urban and rural, heterogeneity, cross-sectional study

## Abstract

**Background:**

Fringe benefits are an important social support in the work scenario, but empirical research on their effect on the mental health of employees is lacking. This study aims to analyse the relationship between different fringe benefits and depression in urban and rural areas.

**Methods:**

Chi-square analysis was used to describe the distribution differences of individual characteristics between urban and rural areas in depression groups. Logistic regression was used to further estimate the relationship between fringe benefits and depression between urban and rural areas.

**Results:**

People with free lunch in urban areas are less likely to suffer from depression. People with food subsidies in rural areas are also less likely to suffer from depression. The abnormal result is people with housing subsidies in rural areas are more likely to be depressed. The effects of job type and contract on depression were different between urban and rural areas.

**Conclusions:**

Employers in urban areas should focus on providing free lunch and the quality of lunch, whereas in rural areas, catering subsidies may be a more appropriate way to improve the mental health of employees. The psychological status of rural workers who receive housing subsidies and have contracts also needs attention.

## Background

Depression is very common worldwide and is a growing public health concern. More than 260 million people are affected, resulting in 43.1 million full age disability adjusted life years (DALY) (1). In China, from 1990 to 2017, the prevalence of depression of all ages per 100,000 increased from 3,224.6 (95%UI: 2,976.6–3,509.1) to 3,990.5 (95%UI: 36,677.8–4,353.0). The DALY rate per 100,000 increased from 525.1 (95%UI: 373.5–719.0) to 607.4 (95%UI: 427.7–820.2) (2).

Social support could have a positive effect on alleviating depression. Depressed patients with poor social support have poor symptoms and rehabilitation results (3). A Chilean study showed that maintaining and improving perceived levels of social support can significantly improve the depression of caregivers (4). A Chinese study also found that people with less social support had more severe depression, and the relationship was mediated by hope (5).

Studies on the relationship between social support and depression focus on the formal support of policies, the social network of individuals and families and the application of specific substances. Disability support pensions in Australia significantly improve the mental health of people with disabilities (6). The expansion policy of Medicaid significantly increases the ability of patients with depression to seek medical services (7). A South Korean study found that diversified and solo-restricted groups had higher levels of depression (8). Higher levels of depression are associated with being unmarried, living alone and lack of family and friend networks (9). An Iranian study found that people who used online social media scored significantly lower on depression (10). However, few studies have focused on the relationship between fringe benefits and depression among workers in general employment scenarios.

In the stable employment situation, the fringe benefits may be related to the mental health of employees. The number of fringe benefits provided by the work unit is one of the basic indicators of work quality (11, 12). In addition, fringe benefits could improve employee job satisfaction (13, 14). Therefore, fringe benefits represent a higher evaluation and recognition of the work quality of the employees by the work unit. Thus, they could alleviate the negative impact of hard work and burnout on mood during the work process. However, the relationship between fringe benefits and depression has not been empirically studied. A comparative study of different groups has not been conducted. The relationship between the content of fringe benefits and depression may be different among regions that greatly differ in terms of economic development level and welfare policy.

In China, great differences exist in the level of economic development, welfare and resource allocation between rural and urban areas. In 2018, the per capita disposable income of urban residents reached 39,251 yuan and that of rural residents was 14,617 yuan, which represents a big difference (15). In addition, rural areas receive less formal government support than urban areas, including pension, welfare and mental health services (16, 17).

This study aims to do the following: (1) analyse the distribution of specific fringe benefits between urban and rural depression groups; and (2) analyse the differences in the relationship between specific fringe benefits and depression in urban and rural areas. This study fills the gap in research on the relationship between social support-fringe benefits and depression in the employed population.

This study contributes to the precise intervention of depression. Firstly, the research on the relationship between fringe benefits and depression is conducive to the rational allocation of work welfare resources and to the alleviation of fatigue and burnout caused by work, thereby improving the happiness of employees. Secondly, the research on China's urban and rural areas has implications for other developing countries and regions to effectively adjust their employee welfare policies.

## Methods

### Data Sources

The data used in this study were obtained from China Health and Retirement Longitudinal Study (CHARLS) targeting the middle-aged and older population (45+ years) in China. The data used in the study came from the China Health and Retirement Longitudinal Survey (CHARLS), a large-scale survey conducted by the National Development Research Institute of Peking University and jointly implemented by the Chinese Social Science Survey Center of Peking University and the Youth League Committee of Peking University. Interdisciplinary survey project. The national baseline survey was carried out in 2011. Survey visits were carried out in 150 counties and 450 communities (villages) in 28 provinces (autonomous regions and municipalities) across the country in 2011, 2013, 2015, and 2018. Due to the large number of samples lost in the longitudinal study (withdraw from the survey). The study used data from the most recent survey in 2018, and a total of 1,891 samples entered our analysis.

### Variables and Definitions

#### Depression

The 10-item (frustrated by small things; hard to concentrate on doing things; feeling down; struggling to do anything; hopeful for the future; feeling scared; poor sleep; feeling very happy; feeling lonely; unable to Continue to live) Center for Epidemiologic Studies Depression Scale (CES-D10) was used to describe the depression symptoms. The responses for CES-D10-based questionnaire were coded on a four-point scale from 0 to 3. Therefore, the individual's depression score range is 0–30; 0 means perfect mental state, whereas 30 reflects the most serious depression symptoms. A CES-D10 score ≥12 the mental health of employees indicates depression (18, 19). Therefore, the study used CES-D10-based binary variable to reflect the individual's depression. The reliability and validity of the scale meet the standards (Cronbach'α = 0.789, KMO = 0.871).

#### Fringe Benefits

Individuals with stable employment were asked *via* questionnaire whether they have the following nine fringe benefits (Free lunch, Free breakfast, Free dinner, Meal allowance, Transportation allowance, Free housing, Housing allowance, Company car and Company shuttle bus). To examine the relationship between the diversity of fringe benefits and depression, these variables were assigned values according to the respondent's “Yes” and “No,” and a binary variable was used to represent the individual's status in each fringe benefit.

#### Control Variable

The SES variables analyzed in this study included gender, age, marital status, education, income, living arrangement and lifestyle. Age was divided into three subgroups, namely, 45–50, 51–60, and 61 years old or older. Education in this study was divided into four levels, namely, primary school or below, junior middle school and senior high school and above. Marital status is defined as a binary variable, which means that married couples who live with or temporarily do not live with their spouse have a spouse, whereas other marital statuses indicate having no spouse. Family income was used to measure the economic status of the respondent, and quintile was used to show their economical level. Based on whether the respondents lived in the same town or city as their children, the respondents' living arrangements were divided into two groups. Smoking and drinking were included in the study and classified by lifestyle of smoking or drinking in the year before the survey.

The state of physical health was represented by chronic disease, disability and self-rated health (SRH). Chronic disease variable was divided into three groups, namely, no chronic disease, only one chronic disease and two or more chronic diseases. Self-perceived health is divided into four groups, namely, very good, good, fair, poor or very poor. Disabilities were divided into two groups based on whether or not they answered “yes” in the ADL (dressing, bathing, eating, getting into and out of bed and toileting and controlling urination and defecation) survey. Based on whether the respondents lived in the same town or city as their children, the respondents' living arrangements were divided into two groups.

To control the confounding effects of job-related variables, the study also included the type of work unit, the position of the employee, the professional and technical level of the employee, the presence or absence of contract, the form of wage payment and job satisfaction as the control variables. The type of work unit is divided into four groups, as follows: government, public institutions or NGO; Firm; Individual firm; and Individual household. According to whether the employee has leadership position and professional skill level, occupation type and professional skill are divided into dichotomous variables. Salary payment method was divided into two categories, namely, monthly salary and other. Job satisfaction is divided into three categories, as follows: completely satisfied and very satisfied; relatively satisfied; not very satisfied; and not at all satisfied.

### Statistical Methods

Chi-square analysis was used to test the differences in respondents' characteristics among depression groups within urban and rural areas. Then, logistic regression on urban and rural areas with depression as the outcome variable was performed to explore the association between fringe benefits and depression.

STATA software 13 was used to execute the Chi-square analysis and logistic regression with statistical significance at *p* < 0.05.

### Ethical Approval

Ethical approval for data collection in CHARLS was obtained from the Biomedical Ethics Review Committee of Peking University (IRB00001052–11015). All interviewees gave written informed consent before recruitment.

## Results

Table 1 describes the basic characteristics of the sample and its distribution among the depressed groups. The following were the most common characteristics in urban and rural areas: male, 51–60 years old, with a spouse, self-rated fair health, no chronic illness, no smoking, drinking, no disability, no leadership position, no professional skills, no employment contract and somewhat job satisfaction. In terms of fringe benefits, the following sample characteristics are more common: with free lunch, no free dinner and breakfast, no meal allowance, no transportation allowance, no housing allowance and no company car and shuttle bus. Gender, education, marital status, SRH, chronic disease, physical disability, contract, job satisfaction, family income and transportation allowance were all significantly different among depression groups within urban and rural areas. Smoking, drinking, type of work unit, occupation type, professional skill and salary payment method were only significant among depression groups within urban areas. Meal allowance was only significant among depression groups within rural areas.

**Table 1:** Sample characteristics and univariate analysis results.

**Variables**	**Urban**				**Rural**			
	**Total**	**Depression**		***p***	**Total**	**Depression**		***p***
		**No**	**Yes**			**No**	**Yes**	
**Gender**				0.001				<0.01
Male	509	451 (88.6)	58 (11.4)		738	603 (81.7)	135 (18.3)	
Female	288	230 (79.9)	589 (20.1)		356	247 (69.4)	109 (30.6)	
**Age**				0.463				0.669
45–50	279	237 (84.9)	42 (15.1)		279	222 (79.6)	57 (20.4)	
51–60	380	330 (86.8)	50 (13.2)		564	435 (77.1)	12 (22.9)	
>60	138	114 (82.6)	24 (17.4)		252	193 (76.6)	59 (23.4)	
**Education**				0.005				<0.01
≤ Elementary school	250	200 (80)	50 (20)		621	451 (72.6)	170 (27.4)	
Junior high school	244	209 (85.7)	35 (14.3)		333	280 (84.1)	53 (15.9)	
≥High school	303	272 (89.8)	31 (10.2)		140	119 (85)	21 (15)	
**Marital status**				0.040				0.013
Partnered	743	640 (86.1)	103 (13.9)		1,013	796 (78.6)	217 (21.4)	
Single	54	41 (75.9)	13 (24.1)		81	54 (66.7)	27 (33.3)	
**Living with children**				0.545				0.087
Yes	371	314 (84.6)	57 (15.4)		593	449 (75.7)	144 (24.3)	
No	426	367 (86.2)	59 (13.8)		501	401 (80)	100 (20)	
**SRH**				<0.01				<0.01
Very good	140	135 (96.4)	5 (3.6)		185	165 (89.2)	20 (10.8)	
Good	171	161 (94.2)	10 (5.8)		150	132 (88)	18 (12)	
Fair	401	330 (82.3)	71 (17.7)		595	468 (78.7)	127 (21.3)	
Poor or very poor	85	55 (64.7)	30 (35.3)		164	85 (51.8)	79 (48.2)	
**Chronic disease**				0.013				0.002
None	482	425 (88.2)	57 (11.8)		686	556 (81)	130 (19)	
One	195	162 (83.1)	33 (16.9)		281	206 (73.3)	75 (26.7)	
Two or more	120	94 (78.3)	26 (21.7)		127	88 (69.3)	39 (30.7)	
**Smoking**				0.024				0.131
Yes	323	287 (88.9)	36 (11.1)		454	363 (80)	91 (20)	
No	474	394 (83.1)	80 (16.9)		640	487 (76.1)	153 (23.9)	
**Drinking**				0.001				0.059
Yes	434	387 (89.2)	47 (10.8)		556	445 (80)	111 (20)	
No	363	294 (81)	69 (19)		538	405 (75.3)	133 (24.7)	
**Disability**				<0.01				<0.01
No	766	662 (86.4)	104 (13.6)		1,021	811 (79.4)	210 (20.6)	
Yes	31	19 (61.3)	12 (38.7)		73	39 (53.4)	34 (46.6)	
**Type of work unit**				<0.01				0.195
Government, public institution or NGO	207	186 (89.9)	21 (10.1)		89	73 (82)	16 (18)	
Firm	264	237 (89.8)	27 (10.2)		294	236 (80.3)	58 (19.7)	
Individual firm	260	213 (81.9)	47 (18.1)		491	380 (77.4)	111 (22.6)	
Individual household	66	45 (68.2)	21 (31.8)		220	161 (73.2)	59 (26.8)	
**Occupation type**				0.046				0.389
No leadership position	624	525 (84.1)	99 (15.9)		942	736 (78.1)	206 (21.9)	
With leadership position	173	156 (90.2)	17 (9.8)		152	114 (75)	38 (25)	
**Professional skill**				0.003				0.278
No technical title	625	522 (83.5)	103 (16.5)		1,039	804 (77.4)	235 (22.6)	
With technical title	172	159 (92.4)	13 (7.6)		55	46 (83.6)	9 (16.4)	
**Contract**				0.026				<0.01
Yes	308	274 (89)	34 (11)		228	198 (86.8)	30 (13.2)	
No	489	407 (83.2)	82 (16.8)		866	652 (75.3)	214 (24.7)	
**Salary payment method**				0.008				0.151
Monthly pay	636	554 (87.1)	82 (12.9)		533	424 (79.5)	109 (20.5)	
Others	161	127 (78.9)	34 (21.1)		561	426 (75.9)	135 (24.1)	
**Job satisfaction**				<0.01				<0.01
Very satisfied	157	140 (89.2)	17 (10.8)		251	200 (79.7)	51 (20.3)	
Somewhat satisfied	528	461 (87.3)	67 (12.7)		703	566 (80.5)	137 (19.5)	
Not satisfied	112	80 (71.4)	32 (28.6)		140	84 (60)	56 (40)	
**Family income**				0.001				<0.01
1	160	123 (76.9)	37 (23.1)		233	156 (67)	77 (33)	
2	174	143 (82.2)	31 (17.8)		227	166 (73.1)	61 (26.9)	
3	147	130 (88.4)	17 (11.6)		197	151 (76.6)	46 (23.4)	
4	169	149 (88.2)	20 (11.8)		219	184 (84)	35 (16)	
5	147	136 (92.5)	11 (7.5)		218	193 (88.5)	25 (11.5)	
**Free lunch**				0.954				0.402
No	321	274 (85.4)	47 (14.6)		324	257 (79.3)	67 (20.7)	
Yes	476	407 (85.5)	69 (14.5)		770	593 (77)	177 (23)	
**Free breakfast**				0.097				0.676
No	553	480 (86.8)	73 (13.2)		627	490 (78.1)	137 (21.9)	
Yes	244	201 (82.4)	43 (17.6)		467	360 (77.1)	107 (22.9)	
**Free dinner**				0.540				0.655
No	535	460 (86)	75 (14)		610	477 (78.2)	133 (21.8)	
Yes	262	221 (84.4)	41 (15.6)		485	373 (76.9)	111 (23.1)	
**Meal allowance**				0.071				0.005
No	630	531 (84.3)	99 (15.7)		1,001	767 (76.6)	234 (23.4)	
Yes	167	150 (89.8)	17 (10.2)		93	83 (89.2)	10 (10.8)	
**Transportation allowance**				0.015				0.032
No	675	568 (84.1)	107 (15.9)		1,044	805 (77.1)	239 (22.9)	
Yes	122	113 (92.6)	9 (7.4)		50	45 (90)	5 (10)	
**Free housing**				0.148				0.583
No	567	491 (86.6)	76 (13.4)		546	428 (78.4)	118 (21.6)	
Yes	230	190 (82.6)	40 (17.4)		548	422 (77)	126 (23)	
**Housing allowance**				0.713				0.403
No	761	651 (85.5)	110 (14.5)		1,074	836 (77.8)	238 (22.2)	
Yes	36	30 (83.3)	6 (16.7)		20	14 (70)	6 (30)	
**Company car**				0.085				0.560
No	780	664 (85.1)	116 (14.9)		1,064	828 (77.8)	236 (22.2)	
Yes	17	17 (100)	0		30	22 (73.3)	8 (26.7)	
**Company shuttle bus**				0.509				0.674
No	737	628 (85.2)	109 (14.8)		1,037	807 (77.8)	230 (22.2)	
Yes	60	53 (88.3)	7 (11.7)		57	43 (75.4)	14 (24.6)	

Table 2 describes the relationship between fringe benefits and other characteristics of the sample and depression. In urban areas, people with free lunches are less likely to be depressed. In rural areas people who have meal allowance are less likely to have depression symptoms. Free breakfast and lunch are not significantly associated with depression in urban and rural areas. A more unusual result is that people in rural areas who receive housing allowance are more likely to be depressed. None of the fringe benefits associated with transportation allowance had a significant relationship with depression. Transportation allowances, free housing, and company shuttle buses are not significantly associated with depression in urban and rural areas.

**Table 2:** Multivariate analysis results of fringe benefits on depression.

	**Urban**	**Rural**
Gender (ref: male)	1.568 (0.828, 2.969)	2.160*** (1.355, 3.443)
**Age (ref: 45–50)**		
51–60	0.975 (0.586, 1.623)	1.292 (0.859, 1.944)
>60	0.966 (0.482, 1.937)	1.185 (0.730, 1.925)
**Education (ref: ≤elementary school)**		
Junior high school	0.849 (0.481, 1.498)	0.597** (0.405, 0.882)
High school	1.093 (0.532, 2.246)	0.657 (0.366, 1.179)
**Marital status (ref: partnered)**		
Single	1.571 (0.703, 3.513)	1.282 (0.734, 2.240)
**Living with children (ref: yes)**		
No	0.807 (0.501, 1.301)	0.931 (0.667, 1.299)
**SRH (ref: very good)**		
Good	1.770 (0.568, 5.513)	1.179 (0.579, 2.402)
Fair	4.490 (1.709, 11.792)***	2.034*** (1.191, 3.471)
Poor or very poor	9.561 (3.259, 28.049)***	5.673*** (3.094, 10.401)
**Chronic disease (ref: none)**		
One	1.376 (0.814, 2.324)	1.254 (0.866, 1.818)
Two or more	1.326 (0.730, 2.407)	1.104 (0.674, 1.810)
**Smoking (ref: yes)**		
No	0.954 (0.525,1.733)	0.833 (0.550,1.262)
**Drinking (ref: yes)**		
No	1.162 (0.707,1.908)	0.844 (0.586,1.215)
**Disability (ref: no)**		
Yes	2.081* (0.882,4.910)	2.298*** (1.324,3.990)
**Type of work unit (ref: government, public institution, or NGO)**		
Firm	0.866 (0.415, 1.810)	1.218 (0.597, 2.487)
Individual firm	1.191 (0.549, 2.582)	1.065 (0.537, 2.114)
Individual household	2.773 (1.101, 6.983)**	0.956 (0.456, 2.006)
**Occupation type (ref: no leadership position)**		
With leadership position	0.720 (0.377, 1.376)	1.528* (0.961, 2.429)
**Professional skill (ref: no technical title)**		
With technical title	0.621 (0.289, 1.333)	1.716 (0.719, 4.095)
**Contract (ref: yes)**		
No	0.969 (0.552, 1.700)	1.647* (0.986, 2.752)
**Salary payment method (ref: monthly pay)**		
Others	1.293 (0.731, 2.288)	1.091 (0.754, 1.579)
**Job satisfaction (ref: very satisfied)**		
Somewhat satisfied	1.020 (0.540, 1.924)	1.008 (0.671, 1.513)
Not satisfied	2.439 (1.142, 5.212)**	2.520*** (1.495, 4.246)
**Family income (ref: lowest)**		
2	0.992 (0.536, 1.837)	0.773 (0.493, 1.211)
3	0.741 (0.363, 1.514)	0.810 (0.495, 1.327)
4	1.114 (0.531, 2.339)	0.595** (0.355, 0.996)
5 (highest)	1.131 (0.460, 2.776)	0.452** (0.253, 0.808)
**Free lunch (ref: no)**		
Yes	0.514 (0.272, 0.971)**	1.031 (0.623, 1.705)
**Free breakfast (ref: No)**		
Yes	1.432 (0.767, 2.671)	0.843 (0.529, 1.344)
**Free dinner (ref: no)**		
Yes	0.762 (0.398, 1.461)	1.050 (0.613, 1.799)
**Meal allowance (ref: no)**		
Yes	0.701 (0.337, 1.461)	0.434** (0.194, 0.974)
**Transportation allowance (ref: no)**		
Yes	0.536 (0.220, 1.303)	0.564 (0.199, 1.601)
**Free housing (ref: no)**		
Yes	1.235 (0.728, 2.094)	1.330 (0.892, 1.983)
**Housing allowance (ref: no)**		
Yes	1.846 (0.635, 5.365)	3.775** (1.225, 11.634)
**Company car (ref: no)**		
Yes		1.038 (0.388, 2.778)
**Company shuttle bus (ref: no)**		
Yes	0.708 (0.279, 1.795)	1.274 (0.630, 2.573)
_cons	0.044*** (0.009, 0.204)	0.064*** (0.019, 2.215)
Obs.	780	1,094
Pseudo *R*^2^	0.167	0.156

*95% confidence intervals are in parenthesis*.

*** *p < 0.01*.

** *p < 0.05*.

* *p < 0.1*.

We also found that in urban areas, people who are employed in individual households are more likely to be depressed, but there was no significant association in rural areas. People with employment contracts in rural areas are more likely to be depressed. These results suggest that there are differences in the factors affecting depression between urban and rural areas, which is worthy of further discussion.

## Discussion

We found that people with free lunch in urban areas were less likely to be depressed. People with food allowance in rural areas were also less likely to be depressed. People with housing allowance in rural areas are more likely to be depressed. The effects of job type and contract on depression were different between urban and rural areas.

Urban and rural economic development and working conditions are different. Compared with rural employees, urban employees may enjoy a higher-quality lunch, which also reduces the mental pressure brought about by the economic burden of life to a certain extent. The quality and value of free lunch in rural areas is limited, so it may not significantly improve the mental state of employees. One study indicates that the satisfaction degree of working meal is related to food quality and service level (20). And the enterprises in urban areas have relatively strict performance and attendance evaluation system. Therefore, the free lunch provided by the work unit can reduce the dining time of employees to reduce the attendance pressure. Furthermore, it also expresses the enterprise's sense of identity to employees. Rural workers who have lunch allowances have more choices when they have more time.

Urban and rural housing system and social environment may be the potential explanatory factors of the relationship between housing allowance and depression in rural areas. Housing subsidy is given by employers to employees who do not have their own housing. China's rural housing system is the homestead system, which means that farmers can build their own houses on their own on collective land (21). On one hand, under this system, most of the farmers have their own houses because of the collective ownership of rural land in China. Therefore, it is rare for people without housing to enjoy a housing allowance. On the other hand, housing allowance cannot fundamentally change the state of no self-owned housing. In rural China, traditional family and neighborhood relationships are the main components of the social network; thus, this kind of social network is limited but stable (22, 23). The information of individual without house may spread in the rural social network, thereby forming an unfriendly social environment. The adverse effect on mental health may exceed the mitigation effect of housing allowance. In China's urban areas, no self-owned housing is generally more popular because of the high degree of marketisation of housing and registered residence. Compared with the high housing prices in urban areas, housing allowance may have limited effect on alleviating the mental problems of workers.

The lack of formal support, such as social welfare, may be responsible for the relationship between work unit type and depression. Individual households are different from individual industrial and commercial households in terms of the types of employers. Individual households comprise informal employers that rely on social relations and rules for governance. They lack formal business registration. Therefore, formal social welfare, such as industrial, commercial and unemployment insurance, cannot be handled for employees. Compared with the social welfare support provided by a large number of formal enterprises in urban areas, this relative mental deprivation will be more intense, thereby aggravating the symptoms of depression. In rural areas, small and medium-sized and small and micro enterprises are the main employment agencies (24). The heterogeneity of employment agencies is small. The gap of relative deprivation is small. Therefore, no significant relationship exists between employment types and depression in rural areas.

Conflict governance mechanism of social relations may be the potential reason for the relationship between contract and depression. Social relations in rural areas in China are social networks composed of acquaintances, such as relatives and neighbors (25). The governance mechanism of implicit contracts, such as trust, verbal promises and private coordination, has become the main way to coordinate interests. Therefore, formal contracts in rural areas dominated by informal governance mechanisms may cause greater mental pressure on employees.

## Limitations

Firstly, the types of fringe benefits in the study include basic life support. In fact, the types of extra benefits may include life support and recreational activities (public travel, public entertainment and paid vacation) and holiday gifts. However, no relevant items are found in the questionnaire. Thus, more research on non-life support is needed. In addition, more samples with more balanced characteristics need to be further analyzed when future data becomes available.

Secondly, this is a cross-sectional study that did not consider the effects of time and longitudinal changes. Although the survey of the data source is a longitudinal survey, a sample with fringe benefits information is missing too much from the longitudinal survey. Moreover, the study only used samples over 45 years old due to the age limit of the survey respondents.

Moreover, the study includes family surveys and excluded high-risk groups, such as those who could not recover from hospitalization for major mental illness. However, this situation also shows that the results are relatively robust.

## Conclusions

The relationship between fringe benefits and depression in urban and rural areas differs. Employees in urban areas who have free lunches are less likely to be depressed. Employees in rural areas who have subsidized meals are less likely to be depressed. People with housing allowance in rural areas are more prone to developing depression. Employers in urban areas need to pay attention to the free provision and quality of lunch, whereas in rural areas, providing catering allowance may be a more appropriate way to improve the mental health of employees. In addition, the mental state of employees who have received housing allowance and have contracts in rural areas needs to become a focus of research.

## Data Availability Statement

The original contributions presented in the study are included in the article/supplementary material, further inquiries can be directed to the corresponding authors.

## Ethics Statement

All procedures performed in this study involving human participants comply with the Ethical Standards of Institutions and/or National Research Committees, as well as the 1964 Helsinki Declaration and its subsequent amendments or similar ethical standards. Ethical approval for data collection in CHARLS was obtained from the Biomedical Ethics Review Committee of Peking University (IRB00001052–11015). All interviewees gave written informed consent before recruitment. The management agency of Peking University's public data approved our data use application.

## Author Contributions

TL wrote the manuscript. CY conceived the idea and provided suggestions for the discussion of the manuscript. XZ critically revised the paper. All authors read and approved the final manuscript.

## Funding

This research was funded by National Science Foundation of China (Grant Number: 72074084). The funding body only provided financial support for the research, and did not participate in any part of the research, such as research design, data analysis, manuscript writing, etc.

## Conflict of Interest

The authors declare that the research was conducted in the absence of any commercial or financial relationships that could be construed as a potential conflict of interest.

## Publisher's Note

All claims expressed in this article are solely those of the authors and do not necessarily represent those of their affiliated organizations, or those of the publisher, the editors and the reviewers. Any product that may be evaluated in this article, or claim that may be made by its manufacturer, is not guaranteed or endorsed by the publisher.

## Abbreviations

CES-D: Center for Epidemiologic Studies Depression Scale
DALYs: Disability adjusted life years
CHARLS: China Health and Retirement Longitudinal Study
SRH: Self-rated health
ADL: Activities of daily living.
